# Global Retinoblastoma Presentation and Analysis by National Income Level

**DOI:** 10.1001/jamaoncol.2019.6716

**Published:** 2020-02-27

**Authors:** Global Retinoblastoma Study Group, Ido Didi Fabian, Elhassan Abdallah, Shehu U. Abdullahi, Rula A. Abdulqader, Sahadatou Adamou Boubacar, Dupe S. Ademola-Popoola, Adedayo Adio, Armin R. Afshar, Priyanka Aggarwal, Ada E. Aghaji, Alia Ahmad, Marliyanti N. R. Akib, Lamis Al Harby, Mouroge H. Al Ani, Aygun Alakbarova, Silvia Alarcón Portabella, Safaa A. F. Al-Badri, Ana Patricia A. Alcasabas, Saad A. Al-Dahmash, Amanda Alejos, Ernesto Alemany-Rubio, Amadou I. Alfa Bio, Yvania Alfonso Carreras, Christiane Al-Haddad, Hamoud H. Y. Al-Hussaini, Amany M. Ali, Donjeta B. Alia, Mazin F. Al-Jadiry, Usama Al-Jumaly, Hind M. Alkatan, Charlotta All-Eriksson, Ali A. R. M. Al-Mafrachi, Argentino A. Almeida, Khalifa M. Alsawidi, Athar A. S. M. Al-Shaheen, Entissar H. Al-Shammary, Primawita O. Amiruddin, Romanzo Antonino, Nicholas J. Astbury, Hatice T. Atalay, La-ongsri Atchaneeyasakul, Rose Atsiaya, Taweevat Attaseth, Than H. Aung, Silvia Ayala, Baglan Baizakova, Julia Balaguer, Ruhengiz Balayeva, Walentyna Balwierz, Honorio Barranco, Covadonga Bascaran, Maja Beck Popovic, Raquel Benavides, Sarra Benmiloud, Nissrine Bennani Guebessi, Rokia C. Berete, Jesse L. Berry, Anirban Bhaduri, Sunil Bhat, Shelley J. Biddulph, Eva M. Biewald, Nadia Bobrova, Marianna Boehme, H.C. Boldt, Maria Teresa B. C. Bonanomi, Norbert Bornfeld, Gabrielle C. Bouda, Hédi Bouguila, Amaria Boumedane, Rachel C. Brennan, Bénédicte G. Brichard, Jassada Buaboonnam, Patricia Calderón-Sotelo, Doris A. Calle Jara, Jayne E. Camuglia, Miriam R. Cano, Michael Capra, Nathalie Cassoux, Guilherme Castela, Luis Castillo, Jaume Català-Mora, Guillermo L. Chantada, Shabana Chaudhry, Sonal S. Chaugule, Argudit Chauhan, Bhavna Chawla, Violeta S. Chernodrinska, Faraja S. Chiwanga, Tsengelmaa Chuluunbat, Krzysztof Cieslik, Ruellyn L. Cockcroft, Codruta Comsa, Zelia M. Correa, Maria G. Correa Llano, Timothy W. Corson, Kristin E. Cowan-Lyn, Monika Csóka, Xuehao Cui, Isac V. Da Gama, Wantanee Dangboon, Anirban Das, Sima Das, Jacquelyn M. Davanzo, Alan Davidson, Patrick De Potter, Karina Q. Delgado, Hakan Demirci, Laurence Desjardins, Rosdali Y. Diaz Coronado, Helen Dimaras, Andrew J. Dodgshun, Craig Donaldson, Carla R. Donato Macedo, Monica D. Dragomir, Yi Du, Magritha Du Bruyn, Kemala S. Edison, I. Wayan Eka Sutyawan, Asmaa El Kettani, Amal M. Elbahi, James E. Elder, Dina Elgalaly, Alaa M. Elhaddad, Moawia M. Ali Elhassan, Mahmoud M. Elzembely, Vera A. Essuman, Ted Grimbert A. Evina, Zehra Fadoo, Adriana C. Fandiño, Mohammad Faranoush, Oluyemi Fasina, Delia D. P. G. Fernández, Ana Fernández-Teijeiro, Allen Foster, Shahar Frenkel, Ligia D. Fu, Soad L. Fuentes-Alabi, Brenda L. Gallie, Moira Gandiwa, Juan L. Garcia, David García Aldana, Pascale Y. Gassant, Jennifer A. Geel, Fariba Ghassemi, Ana V. Girón, Zelalem Gizachew, Marco A. Goenz, Aaron S. Gold, Maya Goldberg-Lavid, Glen A. Gole, Nir Gomel, Efren Gonzalez, Graciela Gonzalez Perez, Liudmira González-Rodríguez, Henry N. Garcia Pacheco, Jaime Graells, Liz Green, Pernille A. Gregersen, Nathalia D. A. K. Grigorovski, Koffi M. Guedenon, D. Sanjeeva Gunasekera, Ahmet K. Gündüz, Himika Gupta, Sanjiv Gupta, Theodora Hadjistilianou, Patrick Hamel, Syed A. Hamid, Norhafizah Hamzah, Eric D. Hansen, J. William Harbour, M. Elizabeth Hartnett, Murat Hasanreisoglu, Sadiq Hassan, Shadab Hassan, Stanislava Hederova, Jose Hernandez, Lorelay Marie Carcamo Hernandez, Laila Hessissen, Diriba F. Hordofa, Laura C. Huang, G. B. Hubbard, Marlies Hummlen, Kristina Husakova, Allawi N. Hussein Al-Janabi, Russo Ida, Vesna R. Ilic, Vivekaraj Jairaj, Irfan Jeeva, Helen Jenkinson, Xunda Ji, Dong Hyun Jo, Kenneth P. Johnson, William J. Johnson, Michael M. Jones, Theophile B. Amani Kabesha, Rolande L. Kabore, Swathi Kaliki, Abubakar Kalinaki, Mehmet Kantar, Ling-Yuh Kao, Tamar Kardava, Rejin Kebudi, Tomas Kepak, Naama Keren-Froim, Zohora J. Khan, Hussain A. Khaqan, Phara Khauv, Wajiha J. Kheir, Vikas Khetan, Alireza Khodabande, Zaza Khotenashvili, Jonathan W. Kim, Jeong Hun Kim, Hayyam Kiratli, Tero T. Kivelä, Artur Klett, Jess Elio Kosh Komba Palet, Dalia Krivaitiene, Mariana Kruger, Kittisak Kulvichit, Mayasari W. Kuntorini, Alice Kyara, Eva S. Lachmann, Carol P. S. Lam, Geoffrey C. Lam, Scott A. Larson, Slobodanka Latinović, Kelly D. Laurenti, Bao Han A. Le, Karin Lecuona, Amy A. Leverant, Cairui Li, Ben Limbu, Quah Boon Long, Juan P. López, Robert M. Lukamba, Livia Lumbroso, Sandra Luna-Fineman, Delfitri Lutfi, Lesia Lysytsia, George N. Magrath, Amita Mahajan, Abdul Rahim Majeed, Erika Maka, Mayuri Makan, Emil K. Makimbetov, Chatonda Manda, Nieves Martín Begue, Lauren Mason, John O. Mason, Ibrahim O. Matende, Miguel Materin, Clarissa C. D. S. Mattosinho, Marchelo Matua, Ismail Mayet, Freddy B. Mbumba, John D. McKenzie, Aurora Medina-Sanson, Azim Mehrvar, Aemero A. Mengesha, Vikas Menon, Gary John V. D. Mercado, Marilyn B. Mets, Edoardo Midena, Divyansh K. C. Mishra, Furahini G. Mndeme, Ahmed A. Mohamedani, Mona T. Mohammad, Annette C. Moll, Margarita M. Montero, Rosa A. Morales, Claude Moreira, Prithvi Mruthyunjaya, Mchikirwa S. Msina, Gerald Msukwa, Sangeeta S. Mudaliar, Kangwa I. Muma, Francis L. Munier, Gabriela Murgoi, Timothy G. Murray, Kareem O. Musa, Asma Mushtaq, Hamzah Mustak, Okwen M. Muyen, Gita Naidu, Akshay Gopinathan Nair, Larisa Naumenko, Paule Aïda Ndoye Roth, Yetty M. Nency, Vladimir Neroev, Hang Ngo, Rosa M. Nieves, Marina Nikitovic, Elizabeth D. Nkanga, Henry Nkumbe, Murtuza Nuruddin, Mutale Nyaywa, Ghislaine Obono-Obiang, Ngozi C. Oguego, Andrzej Olechowski, Scott C. N. Oliver, Peter Osei-Bonsu, Diego Ossandon, Manuel A. Paez-Escamilla, Halimah Pagarra, Sally L Painter, Vivian Paintsil, Luisa Paiva, Bikramjit P. Pal, Mahesh Shanmugam Palanivelu, Ruzanna Papyan, Raffaele Parrozzani, Manoj Parulekar, Claudia R. Pascual Morales, Katherine E. Paton, Katarzyna Pawinska-Wasikowska, Jacob Pe'er, Armando Peña, Sanja Perić, Chau T. M. Pham, Remezo Philbert, David A. Plager, Pavel Pochop, Rodrigo A. Polania, Vladimir G. Polyakov, Manca T. Pompe, Jonathan J. Pons, Daphna Prat, Vireak Prom, Ignatius Purwanto, Ali O. Qadir, Seema Qayyum, Jiang Qian, Ardizal Rahman, Salman Rahman, Jamalia Rahmat, Purnima Rajkarnikar, Rajesh Ramanjulu, Aparna Ramasubramanian, Marco A. Ramirez-Ortiz, Léa Raobela, Riffat Rashid, M. Ashwin Reddy, Ehud Reich, Lorna A. Renner, David Reynders, Dahiru Ribadu, Mussagy M. Riheia, Petra Ritter-Sovinz, Duangnate Rojanaporn, Livia Romero, Soma R. Roy, Raya H. Saab, Svetlana Saakyan, Ahmed H Sabhan, Mandeep S. Sagoo, Azza M. A. Said, Rohit Saiju, Beatriz Salas, Sonsoles San Román Pacheco, Gissela L. Sánchez, Phayvanh Sayalith, Trish A. Scanlan, Amy C. Schefler, Judy Schoeman, Ahad Sedaghat, Stefan Seregard, Rachna Seth, Ankoor S. Shah, Shawkat A. Shakoor, Manoj K. Sharma, Sadik T. Sherief, Nandan G. Shetye, Carol L. Shields, Sorath Noorani Siddiqui, Sidi Sidi Cheikh, Sónia Silva, Arun D. Singh, Niharika Singh, Usha Singh, Penny Singha, Rita S. Sitorus, Alison H. Skalet, Hendrian D. Soebagjo, Tetyana Sorochynska, Grace Ssali, Andrew W. Stacey, Sandra E. Staffieri, Erin D. Stahl, Christina Stathopoulos, Branka Stirn Kranjc, David K. Stones, Caron Strahlendorf, Maria Estela Coleoni Suarez, Sadia Sultana, Xiantao Sun, Meryl Sundy, Rosanne Superstein, Eddy Supriyadi, Supawan Surukrattanaskul, Shigenobu Suzuki, Karel Svojgr, Fatoumata Sylla, Gevorg Tamamyan, Deborah Tan, Alketa Tandili, Fanny F. Tarrillo Leiva, Maryam Tashvighi, Bekim Tateshi, Edi S. Tehuteru, Luiz F. Teixeira, Kok Hoi Teh, Tuyisabe Theophile, Helen Toledano, Doan L. Trang, Fousseyni Traoré, Sumalin Trichaiyaporn, Samuray Tuncer, Harba Tyau-Tyau, Ali B. Umar, Emel Unal, Ogul E. Uner, Steen F. Urbak, Tatiana L. Ushakova, Rustam H. Usmanov, Sandra Valeina, Milo van Hoefen Wijsard, Adisai Varadisai, Liliana Vasquez, Leon O. Vaughan, Nevyana V. Veleva-Krasteva, Nishant Verma, Andi A. Victor, Maris Viksnins, Edwin G. Villacís Chafla, Vicktoria Vishnevskia-Dai, Tushar Vora, Antonio E. Wachtel, Werner Wackernagel, Keith Waddell, Patricia D. Wade, Amina H. Wali, Yi-Zhuo Wang, Avery Weiss, Matthew W. Wilson, Amelia D. C. Wime, Atchareeya Wiwatwongwana, Damrong Wiwatwongwana, Charlotte Wolley Dod, Phanthipha Wongwai, Daoman Xiang, Yishuang Xiao, Jason C. Yam, Huasheng Yang, Jenny M. Yanga, Muhammad A Yaqub, Vera A. Yarovaya, Andrey A. Yarovoy, Huijing Ye, Yacoub A. Yousef, Putu Yuliawati, Arturo M. Zapata López, Ekhtelbenina Zein, Chengyue Zhang, Yi Zhang, Junyang Zhao, Xiaoyu Zheng, Katsiaryna Zhilyaeva, Nida Zia, Othman A. O. Ziko, Marcia Zondervan, Richard Bowman

**Affiliations:** 1 International Centre for Eye Health, London School of Hygiene & Tropical Medicine, London, United Kingdom; 2 The Goldschleger Eye Institute, Sheba Medical Center, Tel Hashomer, Tel Aviv University, Tel Aviv, Israel; 3 Ophthalmology Department of Rabat, Mohammed V University, Rabat, Morocco; 4 Aminu Kano Teaching Hospital, Bayero University, Kano, Nigeria; 5 Basra Children’s Specialty Hospital, Basra, Iraq; 6 National Hospital of Niamey, Niamey, Niger; 7 University of Ilorin Teaching Hospital, University of Ilorin, Ilorin, Nigeria; 8 Department of Ophthalmology, University of Port Harcourt Teaching Hospital, Port Harcourt, Nigeria; 9 University of California, San Francisco; 10 Department of Pediatrics, Banaras Hindu University, Varanasi, India; 11 Department of Ophthalmology, College of Medicine, University of Nigeria, Enugu, Nigeria; 12 The Children’s Hospital and the Institute of Child Health, Lahore, Pakistan; 13 RS Dr Wahidin Sudirohusodo, Makassar, Indonesia; 14 The Royal London Hospital, Barts Health NHS Trust, and Moorfields Eye Hospital NHS Foundation Trust, London, United Kingdom; 15 Hawler Medical University, Erbil, Iraq; 16 Zarifa Aliyeva National Center of Ophthalmology, Baku, Azerbaijan; 17 Department of Pediatric Ophthalmology, Hospital Vall d’Hebron, Barcelona, Spain; 18 Pediatric Oncology Unit, Children Welfare Teaching Hospital, College of Medicine, University of Baghdad, Baghdad, Iraq; 19 Philippine General Hospital, University of the Philippines, Manila, Philippines; 20 College of Medicine, King Saud University, Riyadh, Saudi Arabia; 21 Unidad Nacional de Oncología Pediátrica, Guatemala City, Guatemala; 22 Instituto Cubano de Oftalmología Ramón Pando Ferrer, Marianao, Havana, Cuba; 23 University of Parakou, Parakou, Benin; 24 St Damien Pediatric Hospital, Port-au-Prince, Haiti; 25 Department of Ophthalmology, American University of Beirut Medical Center, Beirut, Lebanon; 26 Pediatric Oncology Department, National Oncology Center, Sana’a, Yemen; 27 Pediatric Oncology Department, South Egypt Cancer Institute, Assiut University, Assiut, Egypt; 28 University Hospital Center Mother Theresa, Tirana, Albania; 29 Imam Hussein Cancer Center, Karbala, Iraq; 30 St Erik Eye Hospital, Stockholn, Sweden; 31 Ibn Al Haitham Teaching Eye Hospital, Baghdad, Iraq; 32 Beira Central Hospital, Beira, Mozambique; 33 Tripoli Eye Hospital, University of Tripoli, Tripoli, Libya; 34 Oncology Unit, Child’s Central Teaching Hospital, Baghdad, Iraq; 35 National Eye Center, Cicendo Eye Hospital, Bandung, Indonesia; 36 Bambino Gesù IRCCS Children’s Hospital, Rome, Italy; 37 Department of Ophthalmology, School of Medicine, Gazi University, Ankara, Turkey; 38 Siriraj Hospital, Mahidol University, Bangkok, Thailand; 39 Lighthouse For Christ Eye Centre, Mombasa, Kenya; 40 Department of Ophthalmology, Faculty of Medicine, Ramathibodi Hospital, Mahidol University, Bangkok, Thailand; 41 Yangon Eye Hospital, University of Medicine 1, Yangon, Myanmar; 42 Retina Consultants of Houston, Houston, Texas; 43 Scientific Center of Pediatrics and Pediatric Surgery, Almaty, Kazakhstan; 44 Pediatric Oncology Unit, Hospital Universitario y Politécnico La Fe, Valencia, Spain; 45 Institute of Pediatrics, Jagiellonian University Medical College, Children’s University Hospital of Krakow, Krakow, Poland; 46 Pediatric Hematology-Oncology Unit, Lausanne University Hospital, Lausanne, Switzerland; 47 Hospital Nacional de Niños Dr Carlos Sáenz Herrera, San Jose, Costa Rica; 48 Department of Pediatric Oncology, University Hassan II Fès, Fez, Morocco; 49 Center Hospitalier et Universitaire Ibn Rochd, Casablanca, Morocco; 50 Ophthalmologic Department of the Teaching Hospital of Treichville, Abidjan, Côte d’Ivoire; 51 Children’s Hospital Los Angeles, Keck School of Medicine, University of Southern California, Los Angeles; 52 The Calcutta Medical Research Institute, Kolkata, India; 53 Department of Pediatric Hematology and Oncology, Narayana Health City, Bangalore, India; 54 University of the Witwatersrand, Johannesburg, South Africa; 55 Department of Ophthalmology, Essen University Hospital, University Duisburg-Essen, Essen, Germany; 56 The Filatov Institute of Eye Diseases and Tissue Therapy, Odessa, Ukraine; 57 Department of Ophthalmology and Visual Sciences, University of Iowa, Iowa City; 58 Hospital das Clínicas da FMUSP, São Paulo, Brazil; 59 Centre Hospitalier Universitaire Yalgado Ouédraogo de Ouagadougou, Ouagadougou, Burkina Faso; 60 Institut Hédi Raïs d’Ophtalmologie, Faculté de Médecine, Université Tunis El Manar, Tunis, Tunisia; 61 Etablissement Hospitalière Spécialise Emir Abdelkader CEA Service d’Oncologie Pédiatrique, Oran, Algeria; 62 Solid Tumor Division, Department of Oncology, St. Jude Children’s Research Hospital, Memphis, Tennessee; 63 Cliniques Universitaires Saint-Luc, Brussel, Belgium; 64 Hospital Infantil Manuel de Jesús Rivera, Managua, Nicaragua; 65 Hospital del Niño Dr. Francisco De Icaza Bustamante, Guayaquil, Ecuador; 66 Department of Ophthalmology, Queensland Children's Hospital, Brisbane, Queensland, Australia; 67 Salud Ocular, Ministerio de Salud Publica, Asuncion, Paraguay; 68 Our Lady’s Children’s Hospital, Dublin, Ireland; 69 Institut Curie, Université de Paris Medicine Paris V Descartes, Paris, France; 70 Centro Hospital Universitário de Coimbra, University of Coimbra, Coimbra, Portugal; 71 Hospital Pereira Rossell, Montevideo, Uruguay; 72 Hospital Sant Joan de Déu, Barcelona, Spain; 73 Hospital Garrahan, Buenos Aires, Argentina; 74 NationalScientific and Technical Research Council, CONICET, Buenos Aires, Argentina; 75 Paediatric Ophthalmology Department, Mayo Hospital and College of Allied Visual Sciences, King Edward Medical University, Lahore, Pakistan; 76 Department of Ophthalmic Plastic Surgery, Orbit and Ocular Oncology, PBMA’s H. V. Desai Eye Hospital, Pune, Maharashtra, India; 77 University of Louisville, Louisville, Kentucky; 78 Ocular Oncology Service, Dr Rajendra Prasad Centre for Ophthalmic Sciences, All India Institute of Medical Sciences, New Delhi, India; 79 Eye Clinic, Department of Ophthalmology, University Hospital Alexandrovska, Medical University, Sofia, Sofia, Bulgaria; 80 Muhimbili National Hospital, Dar es Salaam, Tanzania; 81 National Center for Maternal and Children Health of Mongolia, Ulaanbaatar, Mongolia; 82 Department of Ophthalmology, The Children’s Memorial Health Institute, Warsaw, Poland; 83 Starship Children’s Health, Auckland, New Zealand; 84 Institute of Oncology, Prof. Dr Al. Trestioreanu, Bucharest, Romania; 85 Wilmer Eye Institute, Johns Hopkins Medicine, Baltimore, Maryland, and University of Cincinnati College of Medicine, Cincinnati, Ohio; 86 Indiana University Medical Center, Indianapolis; 87 Bustamante Hospital for Children, Kingston, Jamaica; 88 Semmelweis University, Budapest, Hungary; 89 Department of Ophthalmology, Xinhua Hospital, Shanghai Jiao Tong University School of Medicine, Shanghai, China; 90 Quelimane Central Hospital, Quelimane, Mozambique; 91 Department of Ophthalmology, Songklanagarind Hospital, Prince of Songkla University, Songkla, Thailand; 92 Department of Pediatric Hematology-Oncology, Tata Medical Center, Kolkata, India; 93 Ocular Oncology Services, Dr Shroff’s Charity Eye Hospital, New Delhi, India; 94 Cole Eye Institute, Cleveland Clinic, Cleveland, Ohio; 95 Red Cross War Memorial Children’s Hospital and the University of Cape Town, Cape Town, South Africa; 96 National Children’s Hospital, Panama City, Panama; 97 Department of Ophthalmology, Kellogg Eye Center, University of Michigan, Ann Arbor; 98 Institut Curie, Paris, France; 99 Instituto Nacional de Enfermedades Neoplasicas, Lima, Perú; 100 The Hospital for Sick Children, Toronto, Ontario, Canada; 101 Department of Paediatrics, University of Otago, Christchurch, Children’s Haematology and Oncology Center, Christchurch Hospital, Christchurch, New Zealand; 102 The Children’s Hospital at Westmead, Sydney, New South Wales, Australia; 103 Pediatric Oncology Institute, Federal University of São Paulo, São Paulo, Brazil; 104 Department of Ophthalmology, The First Affiliated Hospital of Guangxi Medical University, Nanning, China; 105 University of KwaZulu-Natal, Durban, South Africa; 106 Ophthalmology Department, Dr M. Djamil General Hospital, Faculty of Medicine, Andalas University, West Sumatra, Indonesia; 107 Department of Ophthalmology, Faculty of Medicine, Udayana University, Sanglah Eye Hospital, Bali, Indonesia; 108 Department of Ophthalmology, Royal Children’s Hospital, Parkville, Victoria, Australia; 109 Department of Paediatrics, Melbourne Medical School, University of Melbourne, Parkville, Victoria, Australia; 110 Children’s Cancer Hospital Egypt 57357, Cairo, Egypt; 111 Department of Oncology, National Cancer Institute, University of Gezira, Wadi Madani, Sudan; 112 Ophthalmology Unit, Department of Surgery, School of Medicine and Dentistry, University of Ghana, Accra, Ghana; 113 Magrabi ICO Cameroon Eye Institute, Yaounde, Cameroon; 114 Aga Khan University, Karachi, Pakistan; 115 Pediatric Growth and Development Research Center, Institute of Endocrinology and Metabolism, Iran University of Medical Sciences, Rasool Akram Hospital, Tehran, Iran; 116 Department of Ophthalmology, University College Hospital, University of Ibadan, Ibadan, Nigeria; 117 Mi Clinic, Ciudad del Este, Paraguay; 118 Hospital Universitario Virgen Macarena, Sevilla, Spain; 119 Hadassah Medical Center, Hebrew University of Jerusalem, Jerusalem, Israel; 120 Hospital Escuela, Tegucigalpa, Honduras; 121 Pediatric Oncology Department, Benjamin Bloom National Children’s Hospital, San Salvador, El Salvador; 122 Lions Sight First Eye Hospital, Queen Elizabeth Central Hospital, Blantyre, Malawi; 123 ClínicaAnglo American, Lima, Perú; 124 Servicio Andaluz de Salud, Sevilla, Spain; 125 Charlotte Maxeke Johannesburg Academic Hospital, Johannesburg, South Africa; 126 Retina and Vitreous Service, Farabi Eye Hospital, Tehran University of Medical Sciences, Tehran, Iran; 127 Department of Ophthalmology, School of Medicine, Addis Ababa University, Addis Ababa, Ethiopia; 128 Murray Ocular Oncology and Retina, Miami, Florida; 129 Sackler Faculty of Medicine, Tel Aviv University, Tel Aviv, Israel; 130 Department of Ophthalmology, Sourasky Medical Center Tel Aviv, School of Medicine, Sackler Faculty of Medicine, Tel Aviv University, Tel Aviv, Israel; 131 Department of Ophthalmology, Boston Children’s Hospital and Harvard Medical School, Boston, Massachusetts; 132 Hospital Civil de Guadalajara, Guadalajara, Mexico; 133 Pediatric Oncology Unit, Instituto Regional de Enfermedades Neoplásicas del Sur, Arequipa, Perú; 134 Unidad de Oncologia Ocular Hospital Oncologico Luis Razzetti, Caracas, Venezuela; 135 IAM NOOR Eye Care Programme, Afghanistan; 136 Department of Clinical Genetics and Center for Rare Disorders, Aarhus University Hospital, Aarhus, Denmark; 137 National Cancer Institute, Rio de Janeiro, Brazil; 138 Département de Pédiatrie, CHU Sylvanus Olympio, Université de Lomé, Lomé, Togo; 139 National Cancer Institute, Maharagama, Sri Lanka; 140 Department of Ophthalmology, Ankara University School of Medicine, Ankara, Turkey; 141 Bai Jerbai Wadia Hospital for Children, Mumbai, India; 142 King George’s Medical University, Lucknow, India; 143 Retinoblastoma Referral Center, University of Siena, Siena, Italy; 144 Centre Hospitalier Universitaire Sainte-Justine, University of Montreal, Montréal, Quebec, Canada; 145 The Indus Hospital, Karachi, Pakistan; 146 Hospital Kuala Lumpur, Kuala Lumpur, Malaysia; 147 John A. Moran Eye Center, University of Utah, Salt Lake City; 148 Bascom Palmer Eye Institute, University of Miami Miller School of Medicine, Miami, Florida; 149 Department of Pediatric Ophthalmology and Strabismus, Al Shifa Trust Eye Hospital, Rawalpindi, Pakistan; 150 University Children’s Hospital, Bratislava, Slovakia; 151 Hospital Nacional Guillermo Almenara Irigoyen, Lima, Perú; 152 Pediatric Hematology and Oncology Center, Mohammed V University, Rabat, Morocco; 153 Department of Pediatrics and Child Health, Jimma University Medical Center, Jimma, Ethiopia; 154 Byers Eye Institute, Stanford University, Stanford, California; 155 Emory Eye Center, Atlanta, Georgia; 156 Department of Ophthalmology, Oslo University Hospital, Oslo, Norway; 157 Oncology Unit, Child’s Central Teaching Hospital, Baghdad, Iraq; 158 Institute for Oncology and Radiology of Serbia, Belgrade, Serbia; 159 Pacific International Hospital, Port Moresby, Papua New Guinea; 160 Eye Department, Birmingham Children’s Hospital, Birmingham Women’s and Children’s NHS Foundation Trust, Birmingham, United Kingdom; 161 Fight Against Angiogenesis-Related Blindness Laboratory, Biomedical Research Institute, Seoul National University Hospital, Seoul, Republic of Korea; 162 Clínica Oftalmológica Pasteur, Santiago, Chile; 163 Storm Eye Institute, Medical University of South Carolina, Charleston; 164 Bukavu Eye Clinic, Bukavu Official University, Bukavu, Democratic Republic of the Congo; 165 Operation Eyesight Universal Institute for Eye Cancer, L V Prasad Eye Institute, Hyderabad, India; 166 Department of Ophthalmology, Makerere University College of Health Sciences Kamplala, Uganda; 167 Division of Pediatric Oncology, School of Medicine, Ege University, Izmir, Turkey; 168 Chang Gung Memorial Hospital, Taipei, Taiwan; 169 Ophthalmology Department, Central Children’s Hospital of Georgia, Tbilisi, Georgia; 170 Division of Pediatric Hematology-Oncology, Department of Pediatrics, Cerrahpaşa Faculty of Medicine and Oncology Institute, Istanbul University, Istanbul, Turkey; 171 St. Anne’s University Hospital Brno, Masaryk University, and International Clinical Research Center/St Anna University Hospital, Brno, Czech Republic; 172 Dhaka Medical College Hospital, Dhaka, Bangladesh; 173 Department of Ophthalmology, Postgraduate Medical Institute, Ameer-Ud-Din Medical College, Lahore General Hospital, Lahore, Pakistan; 174 Angkor Hospital for Children, Krong Siem Reap, Cambodia; 175 Duke Eye Center, Duke University Hospital, Durham, North Carolina; 176 Sankara Nethralaya, Chennai, India; 177 Department of Ophthalmology, Seoul National University Hospital, Seoul, Republic of Korea; 178 Ocular Oncology Service, Department of Ophthalmology, Hacettepe University School of Medicine, Ankara, Turkey; 179 Ocular Oncology Service, Department of Ophthalmology, Helsinki University Hospital, University of Helsinki, Helsinki, Finland; 180 East Tallinn Central Hospital, Tallinn, Estonia; 181 Oncologue Pédiatre Responsable d’Unité de Bangui, Bangui, Central African Republic; 182 Children’s Ophthalmology Department, Children’s Hospital of Vilnius, University Hospital Santaros Clinic, Vilnius, Lithuania; 183 Department of Paediatrics and Child Health, Faculty of Medicine and Health Sciences, Stellenbosch University, Stellenbosch, South Africa; 184 Vitreo-Retina Research Unit, Department of Ophthalmology, Chulalongkorn University, Bangkok, Thailand; 185 University Medical Center Hamburg-Eppendorf, Hamburg, Germany; 186 Hong Kong Eye Hospital, Chinese University of Hong Kong, Hong Kong SAR, China; 187 Perth Children’s Hospital, University of Western Australia, Perth, Western Australia, Australia; 188 Clinical Center of Vojvodina, University Eye Clinic, Eye Research Foundation Vidar–Latinović, Novi Sad, Serbia; 189 Division of Ophthalmology, Feinberg School of Medicine, Northwestern University, and Ann & Robert H. Lurie Children’s Hospital of Chicago, Chicago, Illinois; 190 John A. Burns School of Medicine, University of Hawaii, Honolulu, and University of Southern California Roski Eye Institute, Los Angeles; 191 Division of Ophthalmology, Faculty of Health Sciences, University of Cape Town, Cape Town, South Africa; 192 Phoenix Children’s Hospital, Phoenix, Arizona; 193 Affiliated Hospital of Dali University, Dali City, China; 194 Tilganga Institute of Ophthalmology, Kathmandu, Nepal; 195 Singapore National Eye Center, Singapore, Singapore; 196 Ophthalmology Department, Faculty of Medicine, Universidad de Chile, Santiago, Chile; 197 University Clinics of Lubumbashi, University of Lubumbashi, Lubumbashi, Democratic Rrepublic of Congo; 198 Pediatric Hematology/Oncology/Stem Cell Transplantation, Center for Global Health, Children’s Hospital Colorado, University of Colorado, Aurora; 199 Department of Ophthalmology, Dr Soetomo General Hospital, Airlangga University, Surabaya, Indonesia; 200 Okhmatdyt National Children’s Hospital, Kiev, Ukraine; 201 Pediatric Hematology-Oncology Unit, Apollo Center for Advanced Pediatrics, Indraprastha Apollo Hospital, New Delhi, India; 202 Sekuru Kaguvi Eye Unit, Parirenyatwa Group of Hospitals, Harare, Zimbabwe; 203 National Center of Oncology and Hematology, Bishkek, Kyrgyzstan; 204 University of Alabama at Birmingham, Birmingham; 205 Ruharo Eye Centre, Ruharo Mission Hospital, Mbarara, Uganda; 206 Scottish Livingstone Hospital, Molepolole, Botswana; 207 Department of Ocular Oncology, Royal Victorian Eye and Ear Hospital, East Melbourne, Victoria, Australia; 208 Department of Oncology, Hospital Infantil de México Federico Gómez, Mexico City, Mexico; 209 MAHAK Hematology Oncology Research Center, Mahak Hospital, Tehran, Iran; 210 Department of Ophthalmology, Jimma University, Jimma, Ethiopia; 211 Centre for Sight, New Delhi, India; 212 Department of Ophthalmology, University of Padova, Padova, Italy; 213 Sankara Eye Hospital, Bangalore, India; 214 Kilimanjaro Christian Medical Centre, Moshi, Tanzania; 215 Department of Pathology, Faculty of Medicine, University of Gezira, Wad Medani, Sudan; 216 King Hussein Cancer Center, Amman, Jordan; 217 Department of Ophthalmology, Amsterdam UMC, Amsterdam, the Netherlands; 218 Hospital Infantil Dr Robert Reid Cabral, Santo Domingo, Dominican Republic; 219 Service d’Oncologie Pédiatrique de l’Hôpital Aristide le Dantec, Dakar, Senegal; 220 Ministry of Health, Lusaka, Zambia; 221 Jules-Gonin Eye Hospital, Fondation Asile de Aveugles, University of Lausanne, Lausanne, Switzerland; 222 Department of Ophthalmology, Lagos University Teaching Hospital, College of Medicine of the University of Lagos, Lagos, Nigeria; 223 Abii Specialists Hospital, Bamenda, Cameroon; 224 Aditya Jyot Eye Hospital, Mumbai, India; 225 Lokmanya Tilak Municipal General Hospital and Medical College, Mumbai, India; 226 N.N. Alexandrov National Cancer Centre of Belarus, Minsk, Belarus; 227 Cheikh Anta Diop University, Le Dantec Hospital, Dakar, Senegal; 228 Child Health Department, Faculty of Medicine, Diponegoro University, Semarang, Indonesia; 229 Moscow Helmholtz Research Institute of Eye Diseases, Moscow, Russia; 230 Ho Chi Minh Eye Hospital, Ho Chi Minh, Vietnam; 231 Department of Ophthalmology, Calabar Children’s Eye Center, University of Calabar Teaching Hospital, Calabar Nigeria; 232 Chittagong Eye Infirmary and Training Complex, Chittagong, Bangladesh; 233 Arthur Davison Children’s Hospital, Ndola, Zambia; 234 CHU Angondje Cancerologie, Libreville, Gabon; 235 Sue Anschutz-Rodgers Eye Center, University of Colorado School of Medicine, Aurora; 236 Komfo Anokye Teaching Hospital, Kumasi, Ghana; 237 Clínica Alemana de Santiago, Universidad del Desarrollo, Santiago, Chile; 238 National Ophthalmological Institute of Angola, Luanda, Angola; 239 H M Diwan Eye Foundation, and Tata Medical Center, Kolkata, India; 240 Department of Oncology, Yerevan State Medical University, and Pediatric Cancer and Blood Disorders Center of Armenia, Hematology Center after R. H. Yeolyan, Yerevan, Armenia; 241 University of British Columbia, Vancouver, British Columbia, Canada; 242 University Hospital Center Zagreb, Zagreb, Croatia; 243 Vietnam National Institute of Ophthalmology, Ha Noi, Vietnam; 244 Centre Hospitalier Universitaire de Kamenge, Bujumbura, Burundi; 245 Department of Ophthalmology for Children and Adults, Second Faculty of Medicine, Charles University, and Motol University Hospital, Prague, Czech Republic; 246 Fundacion Clinica Valle del Lili, Cali, Colombia; 247 Head and Neck Tumors Department, SRI of Pediatric Oncology and Hematology, N.N. Blokhin Russian Cancer Research Center, Moscow, Russia; 248 Russian Medical Academy of Postgraduate Education, Moscow, Russia; 249 University Eye Hospital Ljubljana, University Medical Center Ljubljana, Ljubljana, Slovenia; 250 Good Shepherd Hospital, Siteki, Swaziland; 251 Sardjito Hospital, Faculty of Medicine, Universitas Gadjah Mada, Yogyakarta, Indonesia; 252 Hiwa Cancer Hospital, Sulaymaniyah, Iraq; 253 Department of Ophthalmology, Eye and Ear, Nose, and Throat Hospital of Fudan University, Shanghai, China; 254 Department of Ophthalmology, Hospital Infantil de Mexico Federico Gómez, Mexico City, Mexico; 255 Centre Hospitalier Universitaire Joseph Ravoahangy Andrianavalona, Antananarivo, Madagascar; 256 Department of Oculoplasty and Ocular Oncology, Ispahani Islamia Eye Institute and Hospital, Dhaka, Bangladesh; 257 Department of Ophthalmology, Davidoff Center for Oncology, Rabin Medical Center, Sackler School of Medicine, Tel Aviv University, Israel; 258 School of Medicine and Dentistry, Korle-Bu Teaching Hospital, University of Ghana, Accra, Ghana; 259 University of Pretoria, Pretoria, South Africa; 260 Federal Medical Center, Yola, Nigeria; 261 Nampula Central Hospital, Nampula, Mozambique; 262 Division of Pediatric Hematology and Oncology, Department of Pediatrics and Adolescent Medicine, Medical University of Graz, Graz, Austria; 263 Children’s Cancer Institute, American University of Beirut Medical Center, Beirut, Lebanon; 264 NIHR Biomedical Research Centre for Ophthalmology, Moorfields Eye Hospital, and UCL Institute of Ophthalmology and London Retinoblastoma Service, Royal London Hospital, London, United Kingdom; 265 Department of Ophthalmology, Faculty of Medicine, Ain Shams University, Cairo, Egypt; 266 Hospital Dr Manuel Ascencio Villarroel, Cochabamba, Bolivia; 267 Pediatric Hemato-Oncology, Hospital Universitario Infantil La Paz, Madrid, Spain; 268 Hospital Solca Quito, Quito, Ecuador; 269 Mahosot Hospital, Vientiane, Laos; 270 Department of Ophthalmology, Rasool Akram Hospital, Tehran, Iran; 271 Department of Pediatrics, All India Institute of Medical Sciences, New Delhi, India; 272 National Institute of Ophthalmology, Dhaka, Bangladesh; 273 East Timor Eye Program, Dili, Timor-Leste; 274 Tata Memorial Hospital, Mumbai, India; 275 Ocular Oncology Service, Wills Eye Hospital, Thomas Jefferson University, Philadelphia, Pennsylvania; 276 Ophthalmology Department, Nouakchott Medical University, Nouakchott, Mauritania; 277 Department of Ophthalmology, Postgraduate Institute of Medical Education and Research, Chandigarh, India; 278 Department of Ophthalmology, Faculty of Medicine, Universitas Indonesia, and Dr Cipto Mangunkusumo National General Hospital, Jakarta, Indonesia; 279 Casey Eye Institute, Oregon Health & Science University, Portland; 280 Mulago National Referral Hospital, Kampala, Uganda; 281 Department of Ophthalmology, University of Washington, Seattle; 282 Centre for Eye Research Australia, University of Melbourne, East Melbourne, Victoria, Australia; 283 Children’s Mercy Hospital, Kansas City, Missouri; 284 Department of Paediatrics and Child Health, University of the Free State, Bloemfontein, South Africa; 285 BC Children’s Hospital, Vancouver, British Columbia, Canada; 286 Pediatra Hemato-Oncologa, Instituto Oncologico del Oriente Boliviano, Santa Cruz de la Sierra, Bolivia; 287 Henan Children’s Hospital, Affiliated Children’s Hospital of Zhengzhou University, Zhengzhou, China; 288 Queen Sirikit National Institute of Child Health, Bangkok, Thailand; 289 Department of Ophthalmic Oncology, National Cancer Center Hospital, Tokyo, Japan; 290 Department of Pediatric Hematology and Oncology, Second Faculty of Medicine, Charles University, Motol University Hospital, Prague, Czech Republic; 291 Africa Institute of Tropical Ophthalmology, Bamako, Mali; 292 Hospital Nacional Edgardo Rebagliati Martins, Lima, Perú; 293 University Eye Clinic, Skopje, Macedonia; 294 National Cancer Center, Dharmais Cancer Hospital, Jakarta, Indonesia; 295 Ophthalmology Department, Federal University of São Paulo, São Paulo, Brazil; 296 Kabgayi Eye Unit, Gitarama, Rwanda; 297 Department of Pediatric Hematology-Oncology, Schneider Children’s Medical Center, Sackler School of Medicine, Tel Aviv University, Tel Aviv, Israel; 298 Pediatric Oncology Service, Gabriel Toure Hospital, Bamako, Mali; 299 Department of Ophthalmology, Faculty of Medicine, Ocular Oncology Service, Istanbul University, Istanbul, Turkey; 300 Université Adam Barka, Abeche, Chad; 301 Division of Pediatric Hematology-Oncology, Department of Pediatrics, Ankara University, Ankara, Turkey; 302 Department of Ophthalmology, Aarhus University Hospital, Aarhus, Denmark; 303 National Cancer Center of Uzbekistan, Tashkent, Uzbekistan; 304 Children’s Clinical University Hospital, Riga, Latvia; 305 Department of Ophthalmology, Medical University of Graz, Graz, Austria; 306 Jos University Teaching Hospital, Jos, Nigeria; 307 National Eye Center Kaduna, Kaduna, Nigeria; 308 Department of Paediatrics, Beijing Tongren Hospital, Capital Medical University, Beijing, China; 309 Department of Surgery, St Jude Children’s Research Hospital, Memphis, Tennessee; 310 Department of Ophthalmology, Chiang Mai University, Chiang Mai, Thailand; 311 Department of Ophthalmology, Faculty of Medicine, Khon Kaen University, Khon Kaen, Thailand; 312 Department of Pediatric Ophthalmology, Guangzhou Children’s Hospital and Guangzhou Women and Children’s Medical Center, Guangzhou Medical University, Guangzhou, China; 313 Kunming Children’s Hospital, Kunming, China; 314 State Key Laboratory of Ophthalmology, Zhongshan Ophthalmic Center, Sun Yat-sen University, Guangzhou, China; 315 Service d’Ophtalmologie, Cliniques Universitaires de Kinshasa, Université de Kinshasa, Kinshasa, Democratic Republic of Congo; 316 Armed Forces Institute of Ophthalmology, Rawalpindi, Pakistan; 317 S.Fyodorov Eye Microsurgery Federal State Institution, Moscow, Russia; 318 Assistante Hospitalo Universitaire, Faculte de Medecine de Nouakchott Medecin Oncopediatre, Center National d’Oncologie, Nouakchott, Mauritania; 319 Department of Ophthalmology, Beijing Children’s Hospital, Capital Medical University, Beijing, China; 320 Department of Ophthalmology, Children’s Hospital, Zhejiang University School of Medicine, Hangzhou, China; 321 Ophthalmology Department, Great Ormond Street Hospital, London, United Kingdom

## Abstract

**Importance:**

Early diagnosis of retinoblastoma, the most common intraocular cancer, can save both a child’s life and vision. However, anecdotal evidence suggests that many children across the world are diagnosed late. To our knowledge, the clinical presentation of retinoblastoma has never been assessed on a global scale.

**Objectives:**

To report the retinoblastoma stage at diagnosis in patients across the world during a single year, to investigate associations between clinical variables and national income level, and to investigate risk factors for advanced disease at diagnosis.

**Design, Setting, and Participants:**

A total of 278 retinoblastoma treatment centers were recruited from June 2017 through December 2018 to participate in a cross-sectional analysis of treatment-naive patients with retinoblastoma who were diagnosed in 2017.

**Main Outcomes and Measures:**

Age at presentation, proportion of familial history of retinoblastoma, and tumor stage and metastasis.

**Results:**

The cohort included 4351 new patients from 153 countries; the median age at diagnosis was 30.5 (interquartile range, 18.3-45.9) months, and 1976 patients (45.4%) were female. Most patients (n = 3685 [84.7%]) were from low- and middle-income countries (LMICs). Globally, the most common indication for referral was leukocoria (n = 2638 [62.8%]), followed by strabismus (n = 429 [10.2%]) and proptosis (n = 309 [7.4%]). Patients from high-income countries (HICs) were diagnosed at a median age of 14.1 months, with 656 of 666 (98.5%) patients having intraocular retinoblastoma and 2 (0.3%) having metastasis. Patients from low-income countries were diagnosed at a median age of 30.5 months, with 256 of 521 (49.1%) having extraocular retinoblastoma and 94 of 498 (18.9%) having metastasis. Lower national income level was associated with older presentation age, higher proportion of locally advanced disease and distant metastasis, and smaller proportion of familial history of retinoblastoma. Advanced disease at diagnosis was more common in LMICs even after adjusting for age (odds ratio for low-income countries vs upper-middle–income countries and HICs, 17.92 [95% CI, 12.94-24.80], and for lower-middle–income countries vs upper-middle–income countries and HICs, 5.74 [95% CI, 4.30-7.68]).

**Conclusions and Relevance:**

This study is estimated to have included more than half of all new retinoblastoma cases worldwide in 2017. Children from LMICs, where the main global retinoblastoma burden lies, presented at an older age with more advanced disease and demonstrated a smaller proportion of familial history of retinoblastoma, likely because many do not reach a childbearing age. Given that retinoblastoma is curable, these data are concerning and mandate intervention at national and international levels. Further studies are needed to investigate factors, other than age at presentation, that may be associated with advanced disease in LMICs.

## Key Points

**Question:**

Is the income level of a country of residence associated with the clinical stage of presentation of patients with retinoblastoma?

**Findings:**

In this cross-sectional analysis that included 4351 patients with newly diagnosed retinoblastoma, approximately half of all new retinoblastoma cases worldwide in 2017, 49.1% of patients from low-income countries had extraocular tumor at time of diagnosis compared with 1.5% of patients from high-income countries.

**Meaning:**

The clinical stage of presentation of retinoblastoma, which has a major influence on survival, significantly differs among patients from low-income and high-income countries, which may warrant intervention on national and international levels.

## Teaser

This cross-sectional analysis reports the retinoblastoma stage at diagnosis across the world during a single year, investigates associations between clinical variables and national income level, and investigates risk factors for advanced disease at diagnosis.

## Introduction

Retinoblastoma, the most common eye cancer of childhood, is fatal if left untreated. Prognosis of patients with retinoblastoma in high-income countries (HICs) has improved over the past 50 years, now reaching a near 100% disease-free survival rate.^1,2,3^ This is attributed to several factors, including (1) creation of specialized referral centers, (2) decoding of the genetic basis of the disease, (3) formation of screening programs, and (4) the introduction of chemotherapy.^4^ In HICs, retinoblastoma is a curable disease, and attention has now shifted to eye salvage^5,6^ and improvement of quality of life.^7^ In low- and middle-income countries (LMICs), where more than 80% of global retinoblastoma cases arise, the prognosis is poor, and it is assumed that this is because of delayed diagnosis and treatment.^8,9,10^ Publications from LMICs are scarce, and many countries do not report their retinoblastoma data.^11^ The stage of retinoblastoma at the time of diagnosis in low-income, middle-income, and high-income countries has not been surveyed globally. This information is important for policy and health care planning at national and international levels.

The objectives of this study are to (1) report the stage at diagnosis in a large global sample of patients with retinoblastoma, (2) examine associations between clinical variables at presentation and national-income level, and (3) investigate risk factors for advanced disease at diagnosis.

## Methods

This study originated from a consortium of retinoblastoma treatment centers in 8 countries on 3 continents.^12^ From June 2017 through December 2018, all known retinoblastoma treatment centers across the world were contacted by means of personal communications, presentations at scientific conferences, and linking to professional societies in the fields of ophthalmology and oncology to form a global network. All centers involved in the diagnosis and treatment of patients with retinoblastoma, at least by means of enucleation, were eligible to participate.

### Study Design

This study adheres to the Strengthening the Reporting of Observational Studies in Epidemiology (STROBE) reporting guidelines.^13^ It was a 1-year cross-sectional analysis that included all treatment-naive patients with retinoblastoma who presented to participating centers from January 1, 2017, to December 31, 2017, and who were treated or offered treatment for retinoblastoma. A predesigned form was used for data collection (eTable 1 in the Supplement). The data collected included country of residence, sex, first ocular symptom as noted by parents, age at first indication of symptom, age and ocular indication at presentation to the retinoblastoma treatment center, laterality, familial history of retinoblastoma, staging according to the *American Joint Committee on Cancer Staging Manual, Eighth Edition*^14^ and the International Retinoblastoma Staging System,^15^ and primary treatment. Data on country of residence, sex, and laterality were minimum criteria for patient enrollment. The staging classifications were simplified to include only the major subcategories (eTable 2 in the Supplement). For the primary tumor site (cT), the eye with the more advanced disease was used for analysis. Completed forms were electronically uploaded onto a secure server, after which a data quality assurance process was performed (eMethods in the Supplement).

The study was approved by the institutional review board at the London School of Hygiene & Tropical Medicine, which granted a waiver of patient informed consent. Participating centers applied for and received ethics clearance in their countries according to local institutional guidelines.

### Statistical Analysis

All analyses were performed using R software, version 3.5.2 (R Foundation for Statistical Computing), and IBM SPSS Statistics, version 25.0 (IBM Corp). The crude birth rate, country population size, and country classification by national income level were obtained from the 2017 World Population Prospects.^16^ The predicted number of new patients with retinoblastoma per country was calculated as follows: [country population × crude birth rate/1000/17 000], and predicted number per national income level was the sum result of all countries at the same level.

Unless otherwise indicated, summary statistics are presented as median and interquartile range (25%-75%). The *t* test was used to compare means of normally distributed continuous variables, Fisher exact and Pearson χ^2^ tests were used to compare categorical variables, Spearman rank correlation test was used for nonnormal continuous and ordinal variables, and the Cochran-Armitage test^17,18^ was used to test for trend in the proportions of patients with a given parameter across the income levels. Binomial logistic regression was used to model the effect of income level (upper-middle–income level and high-income level combined), presentation age (grouped by tertiles), familial retinoblastoma history, sex, and bilaterality, on the likelihood of children having advanced disease (cT4) at presentation. An α level of .05 and 2-tailed *P* values were used to determine statistical significance.

## Results

The study sample included 4351 treatment-naive patients with retinoblastoma residing in 153 countries (Figure). The data analyzed by national income level are shown in Table 1. Country-level and continent-level data are shown at http://globalretinoblastoma.org (password: Ret2017).

**Figure.**
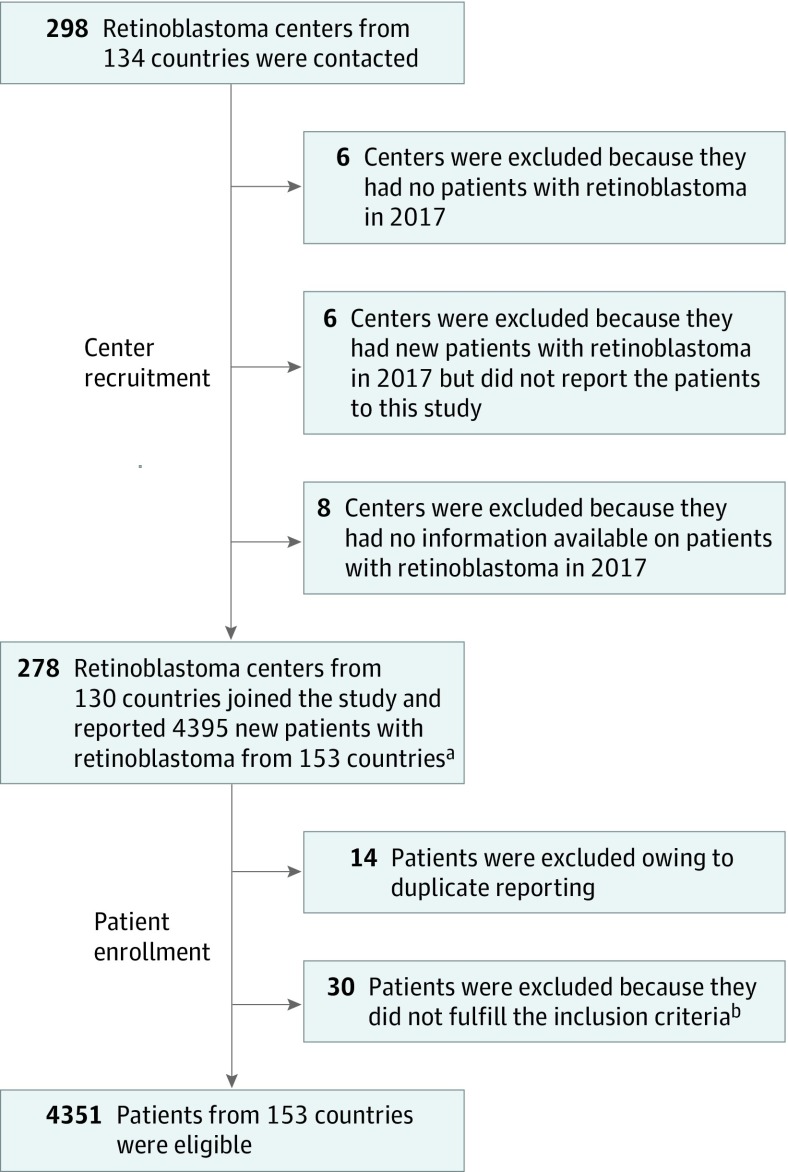
Cohort Recruitment Flowchart ^a^Patients from 23 countries with no retinoblastoma centers were treated outside of their country of residence. ^b^Inclusion criteria included reporting of country of residence, sex, and laterality. Patients for whom 1 or more of these parameters were not available were not included in the analytic sample.

**Table 1.** Clinical Characteristics at Presentation of 4351 New Patients With Retinoblastoma Diagnosed in 2017

Parameter	National Income Level, No. (% within the national income level) [% within the evaluated parameter]				Total, No. (%)	Significance	*P* Value
	Low	Lower-Middle	Upper-Middle	High			
Age at diagnosis, median (IQR), mo							
Total sample	30.5 (18.3-45.9)	24.4 (12.2-37.3)	20.7 (10.1-33.8)	14.0 (6.2-26.6)	23.5 (11.2-36.5)	ρ: −0.22	<.001^a^
Unilateral	35.0 (22.2-48.0)	29.1 (18.1-42.9)	25.5 (12.9-37.6)	19.7 (9.0-32.4)	27.1 (15.0-41.0)		
Bilateral	22.9 (11.8-32.8)	14.4 (8.0-25.8)	11.4 (6.0-21.0)	8.1 (3.7-15.8)	12.3 (6.1-24.3)		
Reported cases, No. (%)	524/533 (98.3)	1909/1940 (98.4)	1192/1212 (98.3)	664/666 (99.7)	4289/4351 (98.6)		
Laterality at diagnosis^b^							
Unilateral	408 (76.5) [13.6]	1325 (68.3) [44.0]	847 (69.9) [28.1]	430 (64.6) [14.3]	3010/4351 (69.2)	NA	<.001^c^
Bilateral	125 (23.5) [9.3]	615 (31.7) [45.9]	365 (30.1) [27.2]	236 (35.4) [17.6]	1341/4351 (30.8)		
Familial history of retinoblastoma							
No	467 (96.9) [11.6]	1805 (96.0) [44.9]	1141 (95.5) [28.4]	603 (91.6) [15.0]	4016/4215 (95.3)	*z* score: −4.3, dim: 4	<.001^d^
Yes	15 (3.1) [7.5]	75 (4.0) [37.7]	54 (4.5) [27.1]	55 (8.4) [27.6]	199/4215 (4.7)		
Total, No. (%)	482/533 (90.4)	1880/1940 (96.9)	1195/1212 (98.6)	658/666 (98.8)	4215/4351 (96.9)		
Primary tumor							
cT1	5 (1.0) [1.8]	96 (5.1) [35.3]	67 (6.1) [24.6]	104 (15.9) [38.2]	272/4114 (6.7)	z score: 22.3, dim: 4	<.001^e^
cT2	62 (12.6) [4.9]	406 (21.7) [31.8]	482 (44.1) [37.8]	326 (49.7) [25.5]	1276/4114 (31.0)		
cT3	209 (42.6) [10.8]	1013 (54.1) [52.4]	488 (44.6) [25.2]	223 (34.0) [11.5]	1933/4114 (47.0)		
cT4	215 (43.8) [34.0]	359 (19.2) [56.7]	56 (5.1) [8.8]	3 (0.4) [0.4]	633/4114 (15.4)		
Total, No. (%)	491/533 (92.1)	1874/1940 (96.6)	1093/1212 (90.2)	656/666 (98.5)	4114/4351 (94.6)		
Regional lymph node							
NX	105 (20.7) [12.6]	350 (18.4) [42.1]	267 (22.2) [32.1]	109 (16.4) [13.1]	831/4281 (19.4)	*z* score: 8.3, dim: 4	<.001^f^
N0	360 (71.0) [10.9]	1475 (77.3) [44.7]	912 (75.9) [27.6]	556 (83.6) [16.8]	3303/4281 (77.2)		
N1	42 (8.3) [28.6]	82 (4.3) [55.8]	23 (1.9) [15.6]	0	147/4281 (3.4)		
Total, No. (%)	507/533 (95.1)	1907/1940 (98.3)	1202/1212 (99.2)	665/666 (99.8)	4281/4352 (98.4)		
Distant metastasis							
M0	404 (81.1) [10.2]	1749 (91.8) [44.1]	1147 (95.2) [28.9]	664 (99.7) [16.8]	3964/4275 (92.7)	*z* score: 11.9, dim: 4	<.001^g^
cM1	65 (13.1) [30.4]	110 (5.8) [51.4]	39 (3.2) [18.2]	0	214/4275 (5.0)		
pM1	29 (5.8) [29.9]	47 (2.5) [48.5]	19 (1.6) [19.6]	2 (0.3) [2.1]	97/4275 (2.3)		
Total, No. (%)	498/533 (89.1)	1906/1940 (98.2)	1205/1212 (99.4)	666/666 (100)	4275/4351 (98.3)		
Hereditary trait							
HX	360 (72.7) [14.2]	1211 (63.8) [47.9]	736 (61.6) [29.1]	221 (33.4) [8.7]	2528/4250 (59.5)	NA	NA
H0	0	44 (2.3) [17.3]	59 (4.9) [23.2]	151 (22.8) [59.4]	254/4250 (6.0)		
H1	135 (27.3) [9.2]	643 (33.9) [43.8]	400 (33.5) [27.2]	290 (43.8) [19.8]	1468/4250 (34.5)		
Total, No. (%)	495/533 (92.9)	1898/1940 (97.8)	1195/1212 (98.6)	662/666 (99.4)	4250/4351 (97.7)		
Extraocular retinoblastoma							
No	265 (50.9) [7.8]	1393 (73.0) [41.3]	1062 (88.0) [31.5]	656 (98.5) [19.4]	3376/4302 (78.5)	*z* score: 21.8, dim: 4	<.001^h^
Yes	256 (49.1) [27.6]	515 (27.0) [55.6]	145 (12.0) [15.7]	10 (1.5) [1.1]	926/4302 (21.5)		
Total, No. (%)	521/533 (97.7)	1908/1940 (98.4)	1207/1212 (99.6)	666/666 (100)	4302/4351 (98.9)		
International Retinoblastoma Staging System							
Stage 0	44 (8.7) [3.0]	459 (24.2) [31.3]	585 (48.8) [39.9]	378 (56.8) [25.8]	1466/4264 (34.4)	NA	NA
Stage I	170 (33.8) [1.0]	816 (43.0) [47.9]	444 (37.0) [26.1]	272 (40.8) [16.0]	1702/4264 (39.9)		
Stage II	58 (11.5) [27.2]	111 (5.9) [52.1]	40 (3.3) [18.8]	4 (0.6) [1.9]	213/4264 (5.0)		
Stage III	101 (20.1) [26.1]	242 (12.8) [62.5]	41 (3.4) [10.6]	3 (0.5) [0.7]	387/4264 (9.1)		
Stage IV	94 (18.7) [29.9]	157 (8.3) [50.0]	60 (5.0) [19.1]	3 (0.5) [1.0]	314/4264 (7.4)		
NA	36 (7.2) [19.8]	111 (5.9) [61.0]	29 (2.4) [15.9]	6 (0.9) [3.3]	182/4264 (4.3)		
Total, No. (%)	503/533 (94.4)	1896/1940 (97.7)	1199/1212 (98.9)	666/666 (100)	4264/4351 (98.0)		

Abbreviations: IQR, interquartile range; NA, not applicable.

^a^ Spearman rank correlation.

^b^ Inclusion criteria: 100% reporting.

^c^ Fisher exact test for proportion of bilateral cases.

^d^ Cochran-Armitage test for proportion of familial history of retinoblastoma.

^e^ Cochran-Armitage test for proportion of cT3 or greater.

^f^ Cochran-Armitage test for proportion of cases with lymph node involvement.

^g^ Cochran-Armitage test for proportion of cases with distant metastasis.

^h^ Cochran-Armitage test for proportion of cases with extraocular disease.

### Geographic and Socioeconomic Characteristics

More than half (2276 [52.3%]) of the patients were from Asia, 1024 (23.5%) were from Africa, 522 (12.0%) were from Europe, 512 (11.8%) were from the Americas, and 17 (0.4%) were from Oceania. Of all patients, 533 (12.3%) came from low-income countries (LICs), 1940 (44.6%) from lower-middle, 1212 (27.9%) from upper-middle, and 666 (15.3%) from HICs.

### Completeness of Data

For 4116 (94.6%) of the study patients, data were reported on each study parameter, except for age at first ocular symptom of retinoblastoma (2175 [50.0%]; not included in the analysis). Analysis by national income level showed that reporting was nearly complete (≥98.5%) for patients from high-income and upper-middle–income countries, and more than 94.1% and 89.1% for patients from lower-middle–income countries and LICs, respectively.

### Symptoms Leading to Referral

The most common first symptom of disease was leukocoria (n = 2638 [62.8%]), followed by strabismus (n = 429 [10.2%]), with a further 162 (3.9%) patients having a combination of leukocoria and strabismus (eTable 3 in the Supplement). Proptosis was reported in 309 (7.4%) patients. At least 1 symptom of advanced disease (ie, proptosis, swollen eyelids, red eye) was reported in 487 (11.7%) patients. A higher income level was associated with a lower proportion of patients with symptoms of advanced disease (*z* score = 10.9, dim = 4; *P* < .001; additional analysis is provided in eTable 4 in the Supplement).

### Symptoms at Time of Diagnosis at Retinoblastoma Centers

Of all patients, 2998 (70.4%) presented with either leukocoria, strabismus, or a combination of these symptoms (eTable 3 in the Supplement). In LICs, combinations of proptosis, red eye, orbital cellulitis, and extraocular retinoblastoma (ie, advanced disease) were present in 248 (46.7%) patients. Analysis of patients who had only leukocoria and/or strabismus (ie, early disease) as the symptoms noticed by the parents, but who presented to retinoblastoma treatment centers with symptoms of advanced disease, showed a significantly larger proportion coming from LICs (*z* score = 18.4, dim = 4; *P* < .001; additional analysis is provided in eTable 4 in the Supplement).

### Age at Diagnosis

The overall median age at diagnosis was 23.5 months (interquartile range [IQR], 11.2-36.5 months; Table 1). The median age at diagnosis of patients from LICs was 30.5 months (IQR, 18.3-45.9 months) compared with 14.0 months (IQR, 6.2-26.6 months) for patients from HICs. There was a significant association between presentation age and national income level, with children in LMICs presenting at an older age (eTable 5 in the Supplement).

### Tumor Staging

Globally, the most common cTNM stages were cT3 (n = 1933 of 4114 [47.0%]), N0 (n = 3303 of 4281 [77.2%]), and M0 (n = 3964 of 4275 [92.7%]) (Table 1). Extraocular retinoblastoma at time of diagnosis was reported in 926 of 4302 (21.5%) patients (256 [49.1%] in LICs vs 10 [1.5%] in HICs). Distant metastases were reported in 94 (18.9%), 157 (8.3%), 58 (4.8%), and 2 (0.3%) patients from low, lower-middle, upper-middle, and high income–level countries, respectively (*z* score = 11.9, dim = 4; *P* < .001). Higher economic grouping was associated with higher proportions of intraocular and earlier stage disease at diagnosis (Table 1).

### Risk Factors for Advanced Disease at Time of Diagnosis

Sex (χ^2^_1_ = 1.016; *P* = .31), bilaterality (χ^2^_1_ = 0.830; *P* = .36) and familial history of retinoblastoma (χ^2^_1_ = 2.269; *P* = .13) were found to be nonsignificant factors for the prediction of cT4 category (extraocular retinoblastoma) and hence were removed from the model. On logistic regression, low-income level and older presentation age were found to be independent and significant predictive factors for advanced disease (Table 2).

**Table 2.** Logistic Regression Analysis: Predictors of Advanced Disease at Presentation^a^^,^^b^

Variable	B (SE)	Corrected *P* Value	Odds Ratio (95% CI)
Income level			
Low vs (upper-middle + high)	2.886 (0.166)	<.001	17.92 (12.94-24.80)
Low-middle vs (upper-middle + high)	1.748 (0.148)	<.001	5.74 (4.30-7.68)
Age at diagnosis			
14.27-31.20 mo	1.343 (0.167)	<.001	3.83 (2.76-5.31)
>31.20 mo	2.026 (0.160)	<.001	7.58 (5.54-10.38)
Constant	−4.602 (0.190)	<.001	0.01

^a^ The logistic regression model was statistically significant (χ^2^_4_ = 727.27; *P* < .001). The model explained 28.5% (Nagelkerke *R*^2^) of the variance and correctly classified 85.1% of cases. Area under the curve was 0.813.

^b^ Advanced disease is defined as cT4.

### Familial History and Bilateral Retinoblastoma

Familial history of retinoblastoma was reported in 199 of 4215 (4.7%) patients (15 [3.1%], 75 [4.0%], 54 [4.5%], and 55 [8.4%] patients from low, lower-middle, upper-middle, and high income–level countries, respectively). Bilateral disease at time of diagnosis was seen in 1341 of 4351 (30.8%) patients (125 [23.5%], 615 [31.7%], 365 [30.1%], and 236 [35.4%] patients from low, lower-middle, upper-middle, and high income–level countries, respectively) (Table 1). Significantly more familial (*z* score = −4.3, dim = 4; *P* < .001) and, independently, more bilateral cases were seen in HICs compared with LICs.

### Diagnostic Facilities and Treatment Modalities

The available diagnostic and treatment modalities are shown in eTable 6 in the Supplement. The majority of patients (4201 [96.6%]) were diagnosed in a center that contained resources for computed tomography and/or magnetic resonance imaging. A histopathology service was available for 4236 (97.4%) participants, and intravenous chemotherapy for 4263 (98.0%).

### Global Magnitude of Retinoblastoma and Representativeness of the Study

Given that the mean age at the time of diagnosis was approximately 2 years old, the 2015 birth rate data were used for calculation of the number of new retinoblastoma cases.^16^ According to these data, the predicted annual number of new retinoblastoma cases worldwide ranged from 7752 to 8914. Using an average incidence figure of 1 of 17 000 live births, capture rates were 88.2%, 56.5%, 48.7%, and 39.9% of expected cases from high, upper-middle, lower-middle, and low-income countries, respectively. No data were received from 65 countries and principalities, mainly with small populations; the estimated number of missing cases from these countries was 46.

## Discussion

Findings of this study show a large disparity in the presentation patterns of retinoblastoma between HICs and LMICs. A total of 666 children were from HICs, 99% of whom had at the time of diagnosis a tumor confined to the eye and thus a favorable prognosis. In comparison, of the 3685 patients from LMICs, 25% were diagnosed with tumor spread beyond the globe, for which the prognosis is much worse.^19,20^ It is likely that the real gap in the pattern of retinoblastoma presentation is even wider owing to unreported patients in LICs who never arrived at a retinoblastoma treatment center and for whom death from metastatic disease is inevitable.

Late cancer diagnosis, also in the pediatric population, is a major issue in LMICs.^21,22,23,24,25^ This study confirms this finding for retinoblastoma, which, if detected early, can be cured. These findings are consistent with a recent study of global disease burden that found that cancer among 0- to 4-year-olds accounts for 37% of the global disease-adjusted life year; this proportional burden is greater in LMICs.^26^

The factors causing delay in retinoblastoma diagnosis and treatment in LMICs are beyond the scope of this study. However, the findings here suggest that late recognition of signs of retinoblastoma, as well as delay in reaching a dedicated retinoblastoma treatment center once ocular symptoms have been detected, likely play a role, and both factors are associated with national income level. These findings indicate clinically significant progression of signs between parental detection and presentation to a specialist center in LMICs. Earlier recognition of leukocoria or strabismus and urgent referral for diagnosis is very important if children are to receive treatment before extraocular spread occurs.

A familial history of retinoblastoma followed the same pattern, with relatively fewer cases in lower-income countries. A possible explanation could be underreporting or inadequate medical record keeping in resource-limited settings. However, a more plausible explanation would be that children with familial history of disease are diagnosed and treated early in HICs so that they survive to childbearing age, whereas this may not be the case in LMICs.

Nearly all essential diagnostic and therapeutic modalities were available in most participating treatment centers. Enucleation surgery, which was available in all treatment centers, can save lives, and intravenous chemotherapy, which was available for 98.0% of the patients in this study, can save lives and also result in globe salvage if patients are diagnosed and treated in time.^27,28^

The results of this study point to an urgent need to improve retinoblastoma detection and access to treatment in LMICs. Several initiatives are addressing this challenge by implementing twinning programs that link centers from higher-resource and lower-resource countries.^12,29,30,31,32^ However, there is a pressing need for coordinated action on a global level. In a rare yet curable cancer such as retinoblastoma, with approximately 8000 new patients annually worldwide, such an action is feasible to make retinoblastoma a zero-death cancer.^33^ The World Health Organization Global Initiative for Childhood Cancer aims to raise survival for key childhood cancers, including retinoblastoma, to 60% by the year 2030 by helping health systems in LMICs integrate childhood cancer into their national strategies and improve their capacity to diagnose and deliver curative treatment.^34^ In this context, accurate retinoblastoma-specific data are essential. The results of this study serve as a report of the current retinoblastoma presentation status, against which future interventions can be measured, and demonstrate the need for a strong global partnership to improve outcomes for patients with retinoblastoma everywhere.

Results of the present study showed that older age at presentation and, independently, national income level were associated with advanced disease, which suggests that other factors besides age may be important in disease progression. It has been suggested that infection by the human papillomavirus, which is more prevalent in LMICs, is associated with the development of nonhereditary retinoblastoma, and it is possible that this could be associated with more aggressive disease behavior.^35^ Another possible explanation relates to the genetic landscape of retinoblastoma and especially to cases with no *RB1* mutation but a high level of amplification of the oncogene *MYCN*.^36^ These cases are unilateral, develop at an early age, and show aggressive features. They were found only in 1.4% of unilateral retinoblastoma cases, all from cohorts in HICs,^36^ but have not been evaluated in patients from LICs. Notably, in the present study, there were substantially more unilateral cases in LICs as compared with other income levels, in keeping with the above-mentioned hypotheses. However, these speculations, warrant further studies.

### Limitations

This study has several limitations. First, it included a convenience sample and therefore had an inherent potential bias. Nevertheless, to our knowledge, it is the largest and most geographically comprehensive study in the field of retinoblastoma, and we believe its findings can be generalized. Second, data collection was mostly retrospective, with the exception of treatment centers that were recruited early in 2017. However, the simplicity of the study design and quality assurance process enabled the collection of almost complete data, also from LMICs. Third, the socioeconomic status of individual families was not included as a variable, and the national income level was used as a surrogate, an approach that assumes that all families from the same country are of the same socioeconomic level.

## Conclusions

The findings of this cross-sectional global analysis of retinoblastoma at the time of diagnosis revealed important differences in presentation among patients from different countries, depending on their national income level. Patients with retinoblastoma from HICs present with early disease and are, therefore, likely to survive. In contrast, patients from lower-income settings present with late disease, many with extraocular extension and some already with metastasis, and their prognosis is poorer. A familial history of retinoblastoma is relatively uncommon in lower-income countries, likely owing to death related to late-disease presentation before childbearing years. A surprise finding of this study is that more advanced disease at presentation in lower-income countries is not entirely explained by older age. Further research is warranted to investigate what factors other than age play a role in disease progression in low-income settings. Prompt action at national and international levels is warranted to improve health education about retinoblastoma, as well as access to early diagnosis and treatment in retinoblastoma treatment centers in LMICs.

## Notes

Supplement. **eMethods.** Data quality assurance **eTable 1.** Sample of the data collection form **eTable 2.** Simplified American Joint Committee on Cancer (AJCC) clinical Tumor, Node, Metastasis, Heredity (cTNMH) and International Retinoblastoma Staging System (IRSS) **eTable 3.** First sign as noticed by parents and presenting sign at retinoblastoma center **eTable 4.** Statistical analysis of clinical variables at presentation by national-income level (Fisher’s exact test) **eTable 5**. Analysis of age at presentation by national-income level (Pearson Chi-square test) **eTable 6.** Available diagnostic facilities and treatment modalities for the study patients
