# Supplementary material for: Global Retinoblastoma Presentation and Analysis by National Income Level
Source: JAMA Oncol. 2020 Feb 27;6(5):1–12. doi: 10.1001/jamaoncol.2019.6716 (PMC7047856; doi:10.1001/jamaoncol.2019.6716)
Supplement: Supplement. — eMethods. Data quality assurance eTable 1. Sample of the data collection form eTable 2. Simplified American Joint Committee on Cancer (AJCC) clinical Tumor, Node, Metastasis, Heredity (cTNMH) and International Retinoblastoma Staging System (IRSS) eTable 3. First sign as noticed by parents and presenting sign at retinoblastoma center eTable 4. Statistical analysis of clinical variables at presentation by national-income level (Fisher’s exact test) eTable 5. Analysis of age at presentation by national-income level (Pearson Chi-square test) eTable 6. Available diagnostic facilities and treatment modalities for the study patients [file jamaoncol-6-685-s001.pdf]

Supplementary Online Content

Global Retinoblastoma Study Group. Global retinoblastoma presentation and analysis by national income level. *JAMA Oncol.* Published online February 27, 2020. doi:10.1001/jamaoncol.2019.6716

eMethods. Data quality assurance

eTable 1. Sample of the data collection form

eTable 2. Simplified American Joint Committee on Cancer (AJCC) clinical Tumor, Node, Metastasis, Heredity (cTNMH) and International Retinoblastoma Staging System (IRSS)

eTable 3. First sign as noticed by parents and presenting sign at retinoblastoma center

eTable 4. Statistical analysis of clinical variables at presentation by national-income level (Fisher's exact test)

eTable 5. Analysis of age at presentation by national-income level (Pearson Chi-square test)

eTable 6. Available diagnostic facilities and treatment modalities for the study patients

This supplementary material has been provided by the authors to give readers additional information about their work.

© 2020 Global Retinoblastoma Study Group. JAMA Oncol.

Section 1. eMethods

Data quality assurance

The data collection form (see eTable 1 below) used by the participating retinoblastoma centers was designed to allow a process of data quality assurance by a third party who has not been exposed to the medical charts. Laterality was reported independently (right, left, or both eyes), as well as under tumor description (T category of cTNMH, reported for each eye separately), IRSS, and primary treatment description. Family history of Rb was reported both independently and as part of the heritable status (H category of cTNMH). Disease staging (i.e., ocular and systemic) was reported under cTNMH and IRSS, and it was required to correlate with the first sign of disease and with the presenting sign at the Rb center. A description of the primary treatment according to the available treatment modalities as reported by centers was required. Four of the authors (IDF, NK, NG and ML) independently checked the completed forms, and the participating centers were contacted and asked for clarifications in the event of inconsistencies. Inadequate data for a specific patient or set of patients was a study exclusion criterion. To avoid duplicate reporting, centers were instructed, a priori, to exclude patients who were diagnosed with Rb and received or were offered treatment in another center. A further measure to ensure that each patient was reported only once included contacting centers in cities with more than one treatment facility (e.g., Moscow, Tehran, Dhaka), with the request that they compare data to detect duplication or submit a joint form. These forms were carefully inspected, and one data entry was removed when there had been duplicate reporting. In a similar manner, the data collection forms submitted by centers from the country of residence of the patients were checked to ensure that patients who travelled across borders for diagnosis and treatment were not reported twice.

Section 2. eTables

eTable 1. Sample of the data collection form.

# of patient	
Rb center (name)	Free text
Rb center (city)	Free text
Rb center (country)	Free text
Distance from home to Rb distance (km)[1]	
Patient country of residence	Free text
Age when first ocular sign was detected by parents/guardians (months)	
Ocular sign as noticed by parent/guardian	Dropdown list: screening for Rb, Incidental finding, leukocoria, strabismus, leukocoria and strabismus, iris heterochromia, loss of visual acuity, proptosis, red eye, swollen eyelids, other (specify in free text)
Age at diagnosis at Rb center (months)	
Ocular sign noticed by care giver in Rb center	Dropdown list: no external sign on evaluation, leukocoria, strabismus, leukocoria and strabismus, proptosis, red eye, orbital cellulitis, extraocular tumor, other (specify in free text)
Sex[1]	Dropdown list: male, female
Rb laterality at time of diagnosis	Dropdown list: right eye, left eye, both eyes
Clinical findings right eye - T of the cTNMH AJCC 8th edition	Dropdown list according the categories shown in eTable 2
Clinical findings left eye - T of the cTNMH AJCC 8th edition	Dropdown list according the categories shown in eTable 2
Regional lymph node involvement – N of the cTNMH AJCC 8th edition	Dropdown list according the categories shown in eTable 2
Specify involved lymph node	Free text
Distant metastasis – M of the cTNMH AJCC 8th edition	Dropdown list according the categories shown in eTable 2
Distant metastasis location	Free text
Heritable trait – H of the cTNMH AJCC 8th edition	Dropdown list according the categories shown in eTable 2

International Retinoblastoma Staging System	Dropdown list according the categories shown in eTable 2
Modalities available in Rb center	Dropdown list: genetic testing, imaging (CT, MRI or CT and MRI), pathology, laser therapy, cryotherapy, intravenous chemotherapy, intra-ophthalmic artery chemotherapy, intra-vitreous chemotherapy, plaque brachytherapy, external beam radiotherapy
Primary treatment given right eye	Dropdown list: laser therapy, cryotherapy, intravenous chemotherapy, intra-ophthalmic artery chemotherapy, plaque brachytherapy, external beam radiotherapy, combination therapy, palliative therapy, treatment refusal
If combination therapy – specify in free text	
Primary treatment given right eye	Dropdown list: laser therapy, cryotherapy, intravenous chemotherapy, intra-ophthalmic artery chemotherapy, plaque brachytherapy, external beam radiotherapy, combination therapy, palliative therapy, treatment refusal
If combination therapy – specify in free text	
[1]not analysed as part of the present manuscript	

eTable 2. Simplified American Joint Committee on Cancer (AJCC) clinical Tumor, Node, Metastasis, Heredity (cTNMH) and International Retinoblastoma Staging System (IRSS).

**AJCC cTNMH**	
**Definition of primary tumor (cT)**	
cTX	Unknown evidence of intraocular tumor
cT0	No evidence of intraocular tumor
cT1	Intraocular tumor(s) with subretinal fluid ≤5 mm from the base of any tumor
cT2	Intraocular tumor(s) with retinal detachment, vitreous seeding, or subretinal seeding
cT3	Advanced intraocular tumor(s), including one or more of the following: phthisis or pre-phthisis bulbi, tumor invasion of the pars plana, ciliary body, lens, zonules, iris, or anterior chamber, raised intraocular pressure with neovascularization and/or buphthalmos, hyphema and/or massive vitreous hemorrhage, or aseptic orbital cellulitis
cT4	Extraocular tumor(s) involving the orbit, including the optic nerve
**Definition of regional lymph nodes (cN)**	
cNX	Regional lymph nodes cannot be assessed
cN0	No regional lymph node involvement
cN1	Evidence of preauricular, submandibular, and cervical lymph node involvement
**Definition of distant metastasis (M)**	
cM0	No signs or symptoms of intracranial or distant metastasis
cM1	Distant metastasis without microscopic confirmation
pM1	Distant metastasis with microscopic confirmation
**Definition of heritable trait (H)**	
HX	Unknown or insufficient evidence of a constitutional *RB*1 gene mutation
H0	Normal *RB*1 alleles in blood tested by high sensitivity assays
H1	Bilateral retinoblastoma, retinoblastoma with an intracranial CNS midline embryonic tumor (trilateral retinoblastoma), family history of retinoblastoma, or molecular definition of constitutional *RB*1 gene mutation
**International Retinoblastoma Staging System**	
Stage 0	Treated conservatively
Stage I	Eye enucleated, completely resected histologically
Stage II	Eye enucleated, microscopic residual tumor
Stage III	Regional extension, including overt orbital disease and/or preauricular or cervical lymph node extension
Stage IV	Metastatic disease, including hematogenous metastasis and/or CNS extension
CNS - central nervous system	

eTable 3. First sign as noticed by parents and presenting sign at retinoblastoma center.

Parameter	National income level (n (%[1], %[2]))				
	Low	Lower-middle	Upper-middle	High	Total
**First sign noticed by parent**					
Screening for Rb	0 (0, 0)	14 (0.8, 17.9)	16 (1.4, 20.5)	48 (7.3, 61.5)	78/4,198 (1.9)
Incidental finding	37 (7.0, 57.8)	5 (0.3, 7.8)	12 (1.0, 18.8)	10 (1.5, 15.6)	64/4,198 (1.5)
Leukokoria	278 (52.4, 10.5)	1,227 (67.2, 46.5)	764 (64.8, 29.0)	369 (55.7, 14.0)	2,638/4,198 (62.8)
Strabismus	15 (2.8, 3.5)	129 (7.1, 30.0)	147 (12.5, 34.3)	138 (20.8, 32.2)	429/4,198 (10.2)
Leukokoria and strabismus	3 (0.6, 1.9)	72 (3.9, 44.4)	53 (4.5, 32.7)	34 (5.1, 21.0)	162/4,198 (3.9)
Iris heterochromia	0 (0, 0)	3 (0.2, 16.7)	7 (0.6, 38.9)	8 (1.2, 44.4)	18/4,198 (0.4)
Loss of visual acuity	14 (2.6, 17.1)	25 (1.4, 30.5)	29 (2.5, 35.4)	14 (2.1, 17.1)	82/4,198 (2.0)
Proptosis	90 (16.9, 29.1)	165 (9.0, 53.4)	48 (4.1, 15.5)	6 (0.9, 1.9)	309/4,198 (7.4)
Red eye	22 (4.1, 17.6)	39 (2.1, 31.2)	57 (4.8, 45.6)	7 (1.1, 5.6)	125/4,198 (3.0)
Swollen eyelids	8 (1.5, 15.1)	33 (1.8, 62.3)	10 (0.8, 18.9)	2 (0.3, 3.8)	53/4,198 (1.3)
Unspecified	64 (12.1, 26.7)	114 (6.2, 47.5)	36 (3.1, 15.0)	26 (3.9, 10.8)	240/4,198 (5.7)
Total	531/533 (99.6)	1,826/1,940 (94.1)	1,179/1,212 (97.3)	662/666 (99.4)	4,198/4,351 (96.5)
**Presenting sign at Rb center**					
No external sign on evaluation	0 (0, 0)	12 (0.6, 17.1)	16 (1.4, 22.9)	42 (6.3, 60.0)	70/4,262 (1.6)
Leukokoria	197 (37.2, 8.1)	1,113 (58.9, 45.7)	734 (62.3, 30.1)	394 (59.5, 16.2)	2,438/4,262 (57.2)
Strabismus	17 (3.2, 5.4)	91 (4.8, 29.0)	112 (9.5, 35.7)	94 (14.2, 30.0)	314/4,262 (7.4)
Leukokoria and strabismus	3 (0.6, 1.2)	101 (5.3, 41.1)	89 (7.5, 36.2)	53 (8.0, 21.5)	246/4,262 (5.8)
Proptosis	191 (36.0, 31.0)	309 (16.3, 50.2)	94 (7.9, 15.3)	22 (3.3, 3.6)	616/4,262 (14.5)
Red eye	9 (1.7, 12.3)	21 (1.1, 28.8)	40 (3.4, 54.8)	3 (0.5, 4.1)	73/4,262 (1.7)
Orbital cellulitis	8 (1.5, 10.5)	45 (2.4, 59.2)	18 (1.5, 23.7)	5 (0.8, 6.6)	76/4,262 (1.8)
Extraocular tumor	40 (7.5, 33.3)	76 (4.0, 63.3)	3 (0.3, 2.5)	1 (0.2, 0.8)	120/4,262 (2.8)
Unspecified	65 (12.3, 21.0)	123 (6.5, 39.8)	73 (6.0, 1.0)	48 (7.3, 15.5)	309/4,262 (7.3)
Total	530/533 (99.4)	1,891/1,940 (97.5)	1,179/1,212 (97.3)	662/666 (99.4)	4,262/4,351 (98.0)

[1]% within the national income level. [2]% within the evaluated parameter.
Rb – retinoblastoma.

eTable 4. Statistical analysis of clinical variables at presentation by national-income level (Fisher's exact test).

Parameter	National-income level	Relation	National-income level	OR	Lower CI	Upper CI	P-value
**Proportion of cases with advanced-disease signs[1] leading to referral**	L	>	LM	2.0	1.5	2.5	<0.001
	L	>	UM	2.7	2.0	3.6	<0.001
	L	>	H	12.5	7.2	23.3	<0.001
	LM	>	UM	1.4	1.1	1.8	0.007
	LM	>	H	6.4	3.8	11.8	<0.001
	UM	>	H	4.7	2.7	8.7	<0.001
**Proportion of cases with early initial signs presenting to center with advanced disease[2]**	L	>	LM	2.7	2.0	3.6	<0.001
	L	>	UM	6.9	4.7	10.3	<0.001
	L	>	H	13.2	7.5	25.0	<0.001
	LM	>	UM	2.6	1.9	3.6	<0.001
	LM	>	H	5.0	2.9	8.9	<0.001
	UM	>	H	1.9	1.1	3.6	0.028
**Proportion of cases with extraocular disease**	L	>	LM	2.6	2.1	3.2	<0.001
	L	>	UM	7.0	5.5	9.1	<0.001
	L	>	H	62.5	33.3	142.9	<0.001
	LM	>	UM	2.7	2.2	3.3	<0.001
	LM	>	H	24.4	13.0	50.0	<0.001
	UM	>	H	8.9	4.7	19.2	<0.001
**Proportion of cases with lymph node**	L	>	LM	2.0	1.3	3.0	0.001
	L	>	UM	4.6	2.7	8.1	<0.001

**involvement**	L	>	H	NA	15.4	NA	<0.001
	LM	>	UM	2.3	1.4	3.9	<0.001
	LM	>	H	NA	7.9	NA	<0.001
	UM	>	H	NA	3.2	NA	<0.001
**Proportion of cases with metastatic spread**	L	>	LM	2.6	1.9	3.4	<0.001
	L	>	UM	4.6	3.2	6.6	<0.001
	L	>	H	76.9	20.4	500.0	<0.001
	LM	>	UM	1.8	1.3	2.5	<0.001
	LM	>	H	29.4	8.1	250.0	<0.001
	UM	>	H	16.7	4.4	142.9	<0.001
**Proportion of cases with familial Rb**	L	<	LM	1.3	0.7	2.4	0.510
	L	<	UM	1.5	0.8	2.8	0.333
	L	<	H	2.8	1.6	5.5	0.001
	LM	<	UM	1.1	08	1.7	0.518
	LM	<	H	2.2	1.5	3.2	<0.001
	UM	<	H	1.9	1.3	2.9	0.003
**Proportion of bilateral Rb cases[3]**	L	<	M	1.5	1.2	1.8	0.001
	L	<	H	1.8	1.4	2.3	<0.001
	M	<	H	1.2	1.0	1.5	0.031

eTable 5. Analysis of age at presentation by national-income level (Pearson Chi-square test).

**National-income level**	**Relation**	**National-income level**	**Age of presentation by tertiles[1, 2]**					
			1st vs 2nd tertile		1st vs 3rd tertile		2nd vs 3rd tertile	
			χ^2	P	χ^2	P	χ^2	P
L	>	LM	5.3	0.02	25.2	<0.001	8.6	0.004
L	>	UM	17.3	<0.001	72.71	<0.001	20.4	<0.001
L	>	H	45.1	<0.001	150.0	<0.001	30.2	<0.001
LM	>	UM	9.3	0.003	29.8	<0.001	6.0	0.016
LM	>	H	44.3	<0.001	108.5	<0.001	15.4	<0.001
UM	>	H	14.5	<0.001	31.2	<0.001	3.9	0.049
[1]1st tertile: age≤14 mo, 2nd tertile: 14 mo<age≤31 mo, 3rd tertile; age>31 mo.<br>[2]for all χ^2 tests, df=1.<br>L - low, LM – lower middle, UM – upper middle, H – high.								

eTable 6. Available diagnostic facilities and treatment modalities for the study patients.[1]

**Parameter**	**National income level (n (%))**				
	**Low (n=533)**	**Lower-middle (n=1,940)**	**Upper-middle (n=1,212)**	**High (n=666)**	**Total (n=4,351)**
Genetic tests	5 (0.9)	402 (20.7)	514 (42.4)	642 (96.4)	1,563 (35.9)
CT	370 (69.4)	435 (22.4)	22 (1.8)	4 (0.6)	831 (19.1)
MRI	88 (16.5)	533 (27.5)	253 (20.9)	231 (34.7)	1,105 (25.4)
CT + MRI	91 (17.1)	899 (46.3)	844 (69.6)	431 (64.7)	2,265 (52.1)
Pathology	501 (94.0)	1,897 (97.8)	1,176 (97.0)	662 (99.4)	4,236 (97.4)
Laser therapy	246 (46.2)	1,615 (83.2)	1,034 (85.3)	659 (98.9)	3,554 (81.7)
Cryotherapy	159 (29.8)	1,299 (67.0)	979 (80.8)	659 (98.9)	3,096 (71.1)
Intravenous chemotherapy	501 (94.0)	1,924 (99.1)	1,172 (96.7)	666 (100)	4,263 (98.0)
Intra-ophthalmic artery chemotherapy	9 (1.7)	596 (30.7)	808 (66.7)	594 (89.2)	2,007 (46.1)
Intravitreal chemotherapy	150 (28.1)	1,076 (55.5)	1,018 (84.0)	657 (98.6)	2,901 (66.7)
Plaque brachytherapy	5 (0.9)	413 (21.3)	232 (19.1)	538 (80.8)	1,188 (27.3)
External beam radiotherapy	213 (40.0)	1,540 (79.4)	798 (65.8)	542 (81.4)	3,093 (71.1)
[1]100% reporting.					
